# Co-Precipitation Synthesis and Optical Properties of Mn^4+^-Doped Hexafluoroaluminate w-LED Phosphors

**DOI:** 10.3390/ma10111322

**Published:** 2017-11-17

**Authors:** Tim Senden, Robin G. Geitenbeek, Andries Meijerink

**Affiliations:** Condensed Matter and Interfaces, Debye Institute for Nanomaterials Science, Utrecht University, P.O. Box 80000, 3508 TA Utrecht, The Netherlands; r.g.geitenbeek@uu.nl (R.G.G.); a.meijerink@uu.nl (A.M.)

**Keywords:** Mn^4+^, red emission, phosphors, white LED, hexafluoroaluminate, thermal quenching

## Abstract

Mn^4+^-activated hexafluoroaluminates are promising red-emitting phosphors for white light emitting diodes (w-LEDs). Here, we report the synthesis of Na_3_AlF_6_:Mn^4+^, K_3_AlF_6_:Mn^4+^ and K_2_NaAlF_6_:Mn^4+^ phosphors through a simple two-step co-precipitation method. Highly monodisperse large (~20 μm) smoothed-octahedron shaped crystallites are obtained for K_2_NaAlF_6_:Mn^4+^. The large size, regular shape and small size distribution are favorable for application in w-LEDs. All Mn^4+^-doped hexafluoroaluminates show bright red Mn^4+^ luminescence under blue light excitation. We compare the optical properties of Na_3_AlF_6_:Mn^4+^, K_3_AlF_6_:Mn^4+^ and K_2_NaAlF_6_:Mn^4+^ at room temperature and 4 K. The luminescence measurements reveal that multiple Mn^4+^ sites exist in M_3_AlF_6_:Mn^4+^ (M = Na, K), which is explained by the charge compensation that is required for Mn^4+^ on Al^3+^ sites. Thermal cycling experiments show that the site distribution changes after annealing. Finally, we investigate thermal quenching and show that the luminescence quenching temperature is high, around 460–490 K, which makes these Mn^4+^-doped hexafluoroaluminates interesting red phosphors for w-LEDs. The new insights reported on the synthesis and optical properties of Mn^4+^ in the chemically and thermally stable hexafluoroaluminates can contribute to the optimization of red-emitting Mn^4+^ phosphors for w-LEDs.

## 1. Introduction

White light emitting diodes (w-LEDs) are nowadays widely applied in general lighting and consumer electronics because of their high energy efficiency and long operation lifetime [1,2,3,4,5]. Commercial w-LEDs generate white light by combining blue-emitting (In,Ga)N LED chips with inorganic phosphors that convert part of the blue LED emission to green, yellow and/or red light [5,6]. Currently, the typical red phosphors in w-LEDs are Eu^2+^-doped nitrides. These phosphors can have quantum yields (QYs) exceeding 90%, but a drawback is that the Eu^2+^ emission band extends into the deep red and near-IR regions where the sensitivity of the human eye is low [6,7,8]. As a result, there are significant efficacy losses (reduced lumen/W output) at high color rendering indices (CRIs) when using Eu^2+^-doped nitrides as red phosphors in w-LEDs [9]. To overcome this issue, efficient narrow band red-emitting phosphors with *λ*_max_ ~ 620 nm have to be developed.

Mn^4+^-doped fluorides are a promising class of materials to improve the color rendering of w-LEDs while maintaining a high luminous efficacy [8,9,10,11]. Upon blue photoexcitation Mn^4+^-doped fluorides show narrow red line emission in the 600–640 nm spectral region. Furthermore, they can have a QY > 90% and are prepared through simple wet-chemical synthesis at room temperature [8,12]. These characteristics make Mn^4+^-doped fluorides interesting narrow band red phosphors for w-LEDs. In recent years, a large number of Mn^4+^-activated fluorides with the general chemical formulas A_2_MF_6_:Mn^4+^ (A = Na, K, Rb, Cs and NH_4_; M = Si, Ge, Ti, Zr and Sn) and BaMF_6_:Mn^4+^ (M = Si, Ge, Ti and Sn) have been reported [11,12,13]. In these compounds Mn^4+^ substitutes for the M^4+^ cation that is octahedrally coordinated by six F^−^ ions. Most work has been done on the phosphor K_2_SiF_6_:Mn^4+^ (KSF) [14,15]. The Mn^4+^-doped fluorides have excellent luminescence properties. The deep red color of the 600–640 nm Mn^4+^ emission is particularly favorable for extending the color gamut of displays, and KSF is already widely applied in displays. In lighting, large-scale application is still limited, partly hampered by issues related to thermal and chemical stability and saturation at high pump powers [14,16].

Recently, the synthesis and luminescence properties of Mn^4+^-doped hexafluoraluminates with the compositions M_3_AlF_6_:Mn^4+^ (M = Li, Na, K) were reported [17,18,19,20]. The ionic radius of Mn^4+^ is similar to the ionic radius of Al^3+^ (0.53 versus 0.54 Å), and as a result, Mn^4+^ can easily substitute for Al^3+^ in fluoride hosts [17,21]. The M_3_AlF_6_:Mn^4+^ phosphors have potential advantages over K_2_SiF_6_:Mn^4+^ and other Mn^4+^-doped fluorides. First, Na_3_AlF_6_ and K_3_AlF_6_ have a melting point of ~1000 °C and therefore have a much better thermal stability than K_2_SiF_6_, which already decomposes at 350–400 °C [10,22,23,24]. Secondly, the M_3_AlF_6_ compounds have a lower water solubility than K_2_SiF_6_, making them more chemically stable under high humidity conditions [14,25]. Thirdly, hexafluoroaluminates are already produced worldwide on a large scale, as they are used as solvents for bauxite in the industrial extraction of aluminum [26]. This may facilitate cheap large-scale production of Mn^4+^-activated hexafluoroaluminates.

Until now, different methods have been used to synthesize M_3_AlF_6_:Mn^4+^ phosphors. Song et al. prepared Na_3_AlF_6_:Mn^4+^ phosphors via a co-precipitation method [17], while others synthesized K_3_AlF_6_:Mn^4+^ and K_2_NaAlF_6_:Mn^4+^ by cation exchange [18,19]. Furthermore, K_2_LiAlF_6_:Mn^4+^ was synthesized via a hydrothermal method [20]. A single convenient synthesis method for preparing M_3_AlF_6_:Mn^4+^ phosphors is thus so far lacking. The previous works on M_3_AlF_6_:Mn^4+^ have reported luminescence spectra and decay curves for different Mn^4+^ doping concentrations in the temperature range of 300 to 500 K [17,18,19,20]. However, they provided no insight into the influence of (charge compensating) defects on the luminescence spectra and quantum yield of M_3_AlF_6_:Mn^4+^. Furthermore, no explanations for the thermal quenching of the Mn^4+^ luminescence were given.

In this work we report a new synthesis route for Na_3_AlF_6_:Mn^4+^, K_3_AlF_6_:Mn^4+^ and K_2_NaAlF_6_:Mn^4+^ based on a simple two-step co-precipitation method. We synthesize Mn^4+^-doped hexafluoroaluminates by initially preparing the Mn^4+^-precursor K_2_MnF_6_ and then in a second step precipitating M_3_AlF_6_:Mn^4+^ (M = Na, K) from an aqueous HF solution containing Al^3+^, Mn^4+^ and Na^+^/K^+^ ions. The presented method gives good control over the composition and homogeneity of the M_3_AlF_6_:Mn^4+^ phosphors. All synthesized M_3_AlF_6_:Mn^4+^ phosphors exhibit bright red Mn^4+^ luminescence around 620 nm. For K_2_NaAlF_6_:Mn^4+^ we obtain highly monodisperse and large (~20 µm) phosphor particles that exhibit narrow size and shape distributions that are superior to the size and shape distributions of other reported Mn^4+^-doped phosphors. This makes K_2_NaAlF_6_:Mn^4+^ interesting for use in w-LEDs, as monodisperse, uniform and large particles are favorable for reproducible and high packing density of phosphors in w-LEDs.

We perform both room-temperature and low-temperature (*T* = 4 K) spectral measurements of M_3_AlF_6_:Mn^4+^. The measurements at 4 K reveal that multiple Mn^4+^ sites exist in M_3_AlF_6_:Mn^4+^, which was not observed in previous works on Mn^4+^-doped hexafluoroaluminates. The formation of multiple Mn^4+^ sites can be understood from the need for charge compensation for Mn^4+^ ions on an Al^3+^ site. Further evidence for the presence of multiple sites is obtained from thermal cycling experiments, which show a change in site distribution after high temperature annealing. The charge compensation and associated defects have a large influence on the luminescence properties (e.g., quantum yield) of M_3_AlF_6_:Mn^4+^.

Finally, we study the thermal quenching behavior for M_3_AlF_6_:Mn^4+^ by measuring the luminescence intensity as a function of temperature between 300 and 600 K. The luminescence quenching temperature we find for M_3_AlF_6_:Mn^4+^ is 460–490 K, which is above the operating temperature of phosphors in high-power w-LEDs. The thermal quenching is explained by thermally activated crossover from the ^4^T_2_ excited state to the ^4^A_2_ ground state. Furthermore, there is luminescence quenching due to non-radiative energy transfer from Mn^4+^ ions to quenching sites (defects and impurities).

## 2. Materials and Methods

The M_3_AlF_6_:Mn^4+^ (M = Na, K) phosphors were synthesized through a two-step chemical co-precipitation method. In the first step the Mn^4+^-precursor K_2_MnF_6_ was synthesized and in the second step the M_3_AlF_6_:Mn^4+^ phosphor was prepared. Since K_2_MnF_6_ and M_3_AlF_6_:Mn^4+^ are synthesized in corrosive HF solutions, all reactions described were carried out in plastic or Teflon beakers.

### 2.1. Chemicals

The following chemicals were purchased from Sigma-Aldrich: KMnO_4_ (≥99.0%), KF (≥99.0%), H_2_O_2_ (30 wt % solution, ACS reagent), Al(OH)_3_ (reagent grade), Na_2_CO_3_ (≥99.95%) and K_2_CO_3_ (≥99.0%). Hydrofluoric acid (HF, 40% aqueous solution) was purchased from Riedel de Haën. All chemicals were used without any further purification.

### 2.2. Synthesis of K_2_MnF_6_

K_2_MnF_6_ was prepared according to the method of Bode [27,28]. KMnO_4_ (4 g) and KF (59.5 g) were dissolved in 250 mL of a 40% HF solution. The black-purple solution obtained was stirred for 30 min and then cooled with an ice bath. Under constant cooling and stirring, a 30 wt % H_2_O_2_ solution was added dropwise which resulted in the gradual precipitation of yellow K_2_MnF_6_ powder. The dropwise addition of H_2_O_2_ was stopped when the reaction solution turned from purple to red-brown, indicating the formation of Mn^4+^. The K_2_MnF_6_ product was isolated by decanting the red-brown solution, washing the precipitate twice with 100 mL of acetone and finally drying the precipitate at 60 °C for 5 h.

### 2.3. Synthesis of M_3_AlF_6_:Mn^4+^

Al(OH)_3_ (10 mmol, 0.78 g) was dissolved in 15 mL 40% HF by stirring and heating at 60 °C. After cooling down to room temperature, 0.1 mmol K_2_MnF_6_ (1 mol % doping concentration) was added. Simultaneously, a solution of M^+^ ions (M = Na, K) in 40% HF was prepared by gradually adding M_2_CO_3_ or MF to 40% HF (aq) while stirring. Table 1 lists the amounts of Na_2_CO_3_, K_2_CO_3_, KF and 40% HF used for preparing the M^+^/HF solutions. The M^+^/HF solution was added to the Al^3+^/Mn^4+^/HF solution, which resulted in the precipitation of Na_3_AlF_6_:Mn^4+^ and K_2_NaAlF_6_:Mn^4+^ but not K_3_AlF_6_:Mn^4+^ phosphor. K_3_AlF_6_:Mn^4+^ was precipitated by adding 50 mL of ethanol to the mixed M^+^/Al^3+^/Mn^4+^/HF solution (ethanol acts as anti-solvent). The synthesized phosphors were isolated by decanting the solution, washing the precipitate twice with ethanol and subsequently drying at 75 °C for 3 h. Different Mn^4+^ doping concentrations could be obtained by changing the amount of K_2_MnF_6_ that was used in the synthesis.

### 2.4. Characterization

Powder X-ray diffraction (XRD) patterns were measured on a Philips PW1729 X-ray diffractometer using Cu K_α_ radiation (*λ* = 1.5418 Å). Scanning electron microscopy (SEM) images of the phosphors were obtained using a Philips XL30S FEG microscope operating at 20 keV. The manganese concentrations in the phosphors were determined with inductively coupled plasma optical emission spectroscopy (ICP-OES) performed on a Perkin-Elmer Optima 8300 spectrometer. For the ICP-OES measurements the M_3_AlF_6_:Mn^4+^ phosphors were dissolved in aqua regia.

Photoluminescence (PL) measurements were performed on an Edinburgh Instruments FLS900 fluorescence spectrometer equipped with a double 0.22 m excitation monochromator. For recording emission and excitation spectra, we used a 450 W Xe lamp as excitation source and a Hamamatsu R928 photomultiplier tube (PMT) to detect the emission. For PL measurements down to 4 K, the phosphors were cooled in an Oxford Instruments liquid helium flow cryostat. PL measurements between 300 K and 600 K were performed by heating the phosphors with a Linkam THMS600 temperature controlled stage. PL quantum yields were determined with a calibrated home-built setup which consisted of a 65 W Xe lamp, excitation monochromator, integrating sphere (Labsphere) and CCD camera (Avantes AvaSpec-2048).

## 3. Results and Discussion

### 3.1. Structural Properties

**Figure 1:**
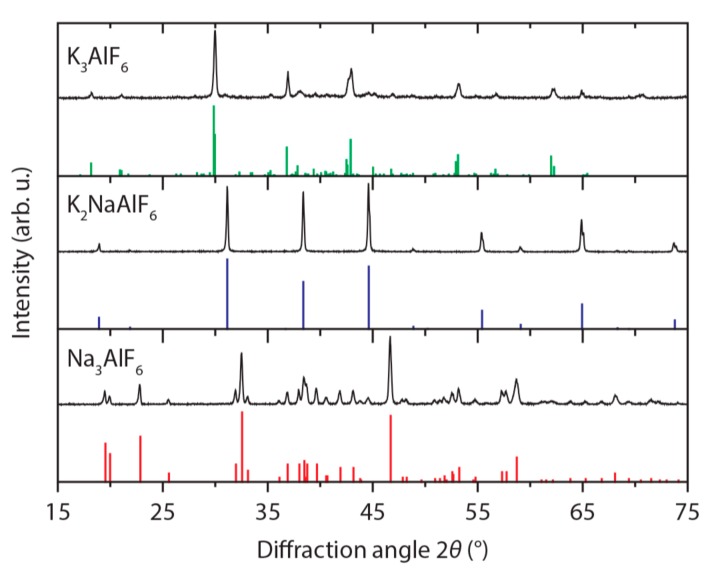
Powder X-ray diffraction (XRD) patterns of M_3_AlF_6_:Mn^4+^ (M = Na, K). The XRD patterns of the synthesized phosphors are in excellent agreement with the reference patterns for Na_3_AlF_6_ (PDF 00-025-0772, red), K_2_NaAlF_6_ (PDF 01-072-2434, blue) and K_3_AlF_6_ (PDF 00-057-0227, green).

To investigate the composition, size and shape of the M_3_AlF_6_:Mn^4+^ (M = Na, K) phosphor particles, we used different characterization techniques such as XRD, SEM and ICP-OES. Figure 1 shows XRD patterns of the M_3_AlF_6_:Mn^4+^ phosphors. The XRD patterns are in perfect agreement with the reference patterns for Na_3_AlF_6_ (PDF 00-025-0772, red), K_2_NaAlF_6_ (PDF 01-072-2434, blue) and K_3_AlF_6_ (PDF 00-057-0227, green). No other crystal phases can be observed, which confirms that the phosphor samples are single phase.

The XRD measurements show that incorporation of Mn^4+^ on the Al^3+^ sites does not significantly change the crystal structure of M_3_AlF_6_, which is expected as the ionic radii of Mn^4+^ and Al^3+^ are similar (0.53 versus 0.54 Å) [21]. The space groups of Na_3_AlF_6_, K_2_NaAlF_6_ and K_3_AlF_6_ are listed in Table 2. K_2_NaAlF_6_ has a highly symmetric cubic crystal structure (space group is *Fm$\bar{3}$m*), while Na_3_AlF_6_ and K_3_AlF_6_ have a crystal structure with lower symmetry (space groups are *P*2_1_/*n* and *I*4_1_/*a*, respectively) [29,30,31]. In the M_3_AlF_6_ crystal structure, the Al^3+^ ions are octahedrally coordinated by six F^−^ ions. Depending on the M_3_AlF_6_ lattice, the AlF_6_ octahedron can be highly symmetric (O_h_ in K_2_NaAlF_6_) or distorted (C_i_ in Na_3_AlF_6_ and C_1_ in K_3_AlF_6_). The average Al–F distances in the AlF_6_ octahedra are around 1.8 Å.

By using ICP-OES, we measured the manganese concentrations in the synthesized M_3_AlF_6_:Mn^4+^phosphors (see Materials and Methods section). The XRD patterns displayed in Figure 1 were measured for M_3_AlF_6_:Mn^4+^ phosphors containing 0.4 mol % (Na_3_AlF_6_:Mn^4+^), 1.2 mol % (K_3_AlF_6_:Mn^4+^) and 0.9 mol % Mn (K_2_NaAlF_6_:Mn^4+^). The results presented in the rest of this work were obtained for M_3_AlF_6_:Mn^4+^ phosphors having these doping concentrations. For K_3_AlF_6_:Mn^4+^ and K_2_NaAlF_6_:Mn^4+^ the measured Mn concentration is very close to the 1 mol % Mn added during the synthesis, which demonstrates that our synthesis method provides good control over the Mn^4+^ doping concentration. Also a substantially higher fraction of Mn^4+^ is incorporated into K_3_AlF_6_ compared to previously reported cation exchange methods for preparing K_3_AlF_6_:Mn^4+^ [18].

**Figure 2:**
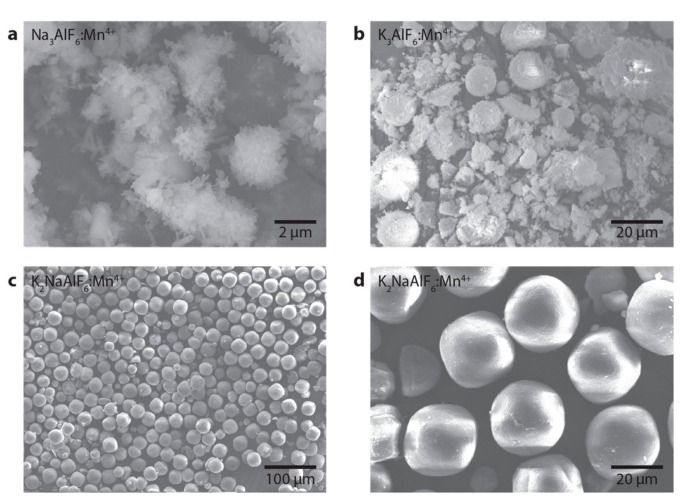
Representative scanning electron microscopy (SEM) images of (**a**) Na_3_AlF_6_:Mn^4+^ (0.4%) phosphor particles; (**b**) K_3_AlF_6_:Mn^4+^ (1.2%) phosphor particles and (**c**,**d**) K_2_NaAlF_6_:Mn^4+^ (0.9%) phosphor particles.

Figure 2 displays SEM images of Na_3_AlF_6_:Mn^4+^ (0.4%), K_3_AlF_6_:Mn^4+^ (1.2%) and K_2_NaAlF_6_:Mn^4+^ (0.9%) phosphor particles. The Na_3_AlF_6_:Mn^4+^ (Figure 2a) and K_3_AlF_6_:Mn^4+^ (Figure 2b) phosphors consist of irregularly shaped particles and clusters of particles with sizes ranging from ~100 nm to >10 µm. For K_3_AlF_6_:Mn^4+^, we attribute the large variety in shape and size to the rapid and forced crystallization following addition of the anti-solvent ethanol. In contrast, the synthesis of K_2_NaAlF_6_:Mn^4+^ (Figure 2c,d) yields highly monodisperse phosphor particles with a large average diameter of 22.5 ± 6.1 µm. The K_2_NaAlF_6_:Mn^4+^ particles have a smoothed octahedral shape, as expected from the cubic elpasolite structure of K_2_NaAlF_6_ [10,19,29]. The K_2_NaAlF_6_:Mn^4+^ particles prepared with our co-precipitation method have a more uniform shape and size than the K_2_NaAlF_6_:Mn^4+^ particles prepared by Zhu et al. via cation exchange [19]. Moreover, the K_2_NaAlF_6_:Mn^4+^ (0.9%) phosphor shown in Figure 2c,d exhibits particle size and shape distributions that are superior to the size and shape distributions of other reported Mn^4+^-doped fluoride phosphors [10,24,32,33,34,35,36,37].

The high monodispersity of the K_2_NaAlF_6_:Mn^4+^ crystallites reported here may originate from the synthesis method used. In order to achieve a narrow size distribution, it is necessary to temporally separate the particle nucleation and growth stages [38]. As described in the Materials and Methods section, we dissolve all the starting materials in HF solutions prior to the formation of the phosphor particles. Mixing of the precursor solutions results in oversaturation and the rapid formation of crystal nuclei. Once the nuclei have been formed, the precursor concentrations drop and no new nuclei are formed. The particles can grow to monodisperse and large crystallites during the growth stage. This differs from syntheses where Mn^4+^-doped particles are prepared via cation exchange or chemical etching [14]. With these methods, new precursor ions are constantly supplied to the reaction solution preventing temporal separation of nucleation and growth.

The superior size distribution gives K_2_NaAlF_6_:Mn^4+^ potential advantages in LED applications. Monodisperse and large crystallites are favorable for uniform and reproducible packing of phosphors, which is very important in the production of w-LEDs. In addition, phosphors with large and highly crystalline particles often display higher quantum yields because of reduced concentrations of defects which act as quenching sites. Finally, a large particle size aids the long-term stability of a phosphor.

### 3.2. Mn^4+^Luminescence

**Figure 3:**
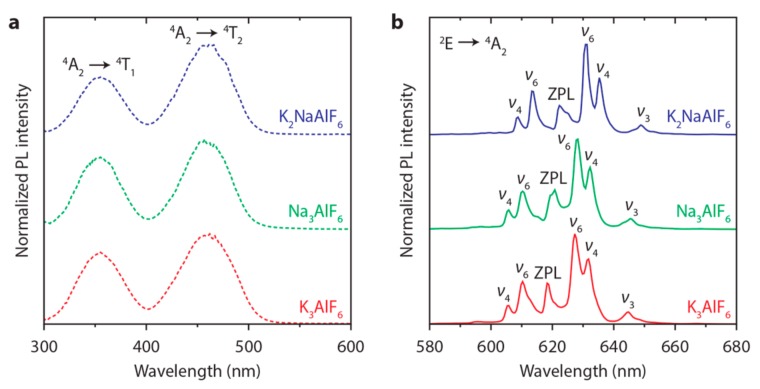
Room-temperature luminescence spectra of M_3_AlF_6_:Mn^4+^ (M = Na, K) phosphors. (**a**) Excitation spectra of K_3_AlF_6_:Mn^4+^ (1.2%) (red, *λ*_em_ = 628 nm), Na_3_AlF_6_:Mn^4+^ (0.4%) (green, *λ*_em_ = 628 nm) and K_2_NaAlF_6_:Mn^4+^ (0.9%) (blue, *λ*_em_ = 631 nm). The broad excitation bands correspond to the Mn^4+^ ^4^A_2_ → ^4^T_1_ and ^4^A_2_ → ^4^T_2_ transitions; (**b**) Emission spectra (*λ*_exc_ = 460 nm) of K_3_AlF_6_:Mn^4+^ (1.2%) (red), Na_3_AlF_6_:Mn^4+^ (0.4%) (green) and K_2_NaAlF_6_:Mn^4+^ (0.9%) (blue). The Mn^4+^emission spectra consist of zero-phonon (ZPL) and (anti-)Stokes vibronic (*ν*_i_) ^2^E → ^4^A_2_ emission lines.

Figure 3 shows the room-temperature emission and excitation spectra of M_3_AlF_6_:Mn^4+^ (M = Na, K). Upon blue photoexcitation the Mn^4+^-doped hexafluoroaluminates show narrow red emission lines around 620 nm (see Figure 3b). The emission lines are in a spectral range where the eye sensitivity is still relatively high, which is beneficial for applications. The red emission lines are assigned to spin- and parity-forbidden Mn^4+^ ^2^E → ^4^A_2_ transitions. Figure 3a shows that the red luminescence of M_3_AlF_6_:Mn^4+^ has two broad excitation bands in the ultraviolet (UV) to blue spectral region. These bands correspond to the ^4^A_2_ → ^4^T_1_ and ^4^A_2_ → ^4^T_2_ transitions of Mn^4+^. In all three lattices the ^4^A_2_ → ^4^T_2_ excitation band is positioned at 460 nm, which indicates that the crystal field splitting is approximately equal for the investigated M_3_AlF_6_ hosts.

The emission spectra of M_3_AlF_6_:Mn^4+^ in Figure 3b resemble the emission spectra observed for other Mn^4+^-doped fluoride phosphors [33,39,40]. By analogy, the ^2^E → ^4^A_2_ emission spectra of M_3_AlF_6_:Mn^4+^ consist of a zero-phonon line (ZPL) at ~620 nm and anti-Stokes and Stokes vibronic (*ν*_3_, *ν*_4_ and *ν*_6_) lines on the high and low energy side of the ZPL, respectively [39]. The Mn^4+^ emission spectra are dominated by vibronic lines because the parity selection rule is relaxed by coupling of the ^2^E → ^4^A_2_ electronic transition with odd-parity vibrations (*ν*_3_, *ν*_4_ and *ν*_6_ vibrational modes) [39,41]. It is noted that the ^2^E → ^4^A_2_ ZPL intensity of M_3_AlF_6_:Mn^4+^ is relatively strong when compared to other Mn^4+^-doped fluorides. For example, in K_2_SiF_6_:Mn^4+^ the ZPL intensity is less than 5% of the Stokes *ν*_6_ intensity, while in K_3_AlF_6_:Mn^4+^ the ZPL intensity is almost half of the Stokes *ν*_6_ intensity [40]. The intense ZPL is an interesting property for (w-LED) applications, as it improves the color quality of the red Mn^4+^ phosphor. Furthermore, the observation of relatively strong zero-phonon lines is a sign that the MnF_6_ centers lack inversion symmetry. The presence of odd-parity crystal field components relaxes the parity selection rule by inducing mixing with opposite parity states. As a result, the radiative lifetime of the ^2^E → ^4^A_2_ emission is shorter (which is beneficial to reduce saturation effects) and the ^4^A_2_ → ^4^T_2_ absorption is stronger (and thus less material or a lower Mn^4+^ concentration is needed to absorb the desired fraction of blue LED light).

The strong ZPL intensity in K_3_AlF_6_:Mn^4+^ can be attributed to the low symmetry for Mn^4+^ on the Al^3+^ sites, i.e., the AlF_6_ octahedron lacks an inversion center (C_1_ symmetry, see Table 2). As discussed, without inversion symmetry, the ^2^E → ^4^A_2_ ZPL intensity increases due to relaxation of the parity selection rule by odd-parity crystal field components that mix odd-parity states into the 3d wavefunctions [42]. In Na_3_AlF_6_ and K_2_NaAlF_6_ the AlF_6_ octahedra have inversion centers (C_i_ and O_h_ symmetry, respectively) and the ^2^E → ^4^A_2_ ZPL is expected to be weak, since there are no odd-parity crystal field components to relax the parity selection rule. The emission spectra in Figure 3b however show that the ZPLs of Na_3_AlF_6_:Mn^4+^ and K_2_NaAlF_6_:Mn^4+^ are significant, which indicates that at least for a part of the Mn^4+^ ions the site symmetry is lower than C_i_ (no inversion symmetry). This we explain by the charge compensation required for the ${\mathrm{Mn}}_{\mathrm{Al}}^{•}$ sites (the Kröger-Vink notation is used to identify defects). The proximity of charge compensating defects (probably $V_{K,\mathrm{Na}}^{\prime }$ vacancies or $F_{i}^{\prime }$ interstitials) gives rise to local deformation of the MnF_6_ octahedra and lifts the inversion symmetry, causing the ^2^E → ^4^A_2_ ZPL intensity of Na_3_AlF_6_:Mn^4+^ and K_2_NaAlF_6_:Mn^4+^to increase.

Besides influencing the ^2^E → ^4^A_2_ ZPL intensity, the charge compensating defects have a large influence on the integrated luminescence intensity and emission lifetime of M_3_AlF_6_:Mn^4+^. Typical luminescence quantum yield (QY) values measured for the M_3_AlF_6_:Mn^4+^ phosphors are 39% for Na_3_AlF_6_:Mn^4+^, 53% for K_2_NaAlF_6_:Mn^4+^ and 55% for K_3_AlF_6_:Mn^4+^. These QY values are below unity, which is attributed to non-radiative transfer of excitation energy from Mn^4+^ to crystal defects. At the defects the excitation energy is lost non-radiatively as heat, i.e., the defects act as quenching sites. The defect concentration will increase with the Mn^4+^ concentration, and it is therefore expected that the luminescence quenching becomes stronger at higher Mn^4+^ concentrations. Previously, it has been measured that the luminescence intensity and emission lifetime of M_3_AlF_6_:Mn^4+^ significantly decrease with increasing Mn^4+^ concentration already at doping levels of 4% Mn^4+^ [17,18,19]. In these works, the decrease in intensity and lifetime with the Mn^4+^ concentration was explained by concentration quenching, i.e., energy migration between Mn^4+^ ions to quenchers (defects, impurities). Energy migration is however not expected at doping concentrations of 4%, as most Mn^4+^ ions will not have Mn^4+^ neighbors in this concentration range (see also [43]). The results presented here indicate that quenching becomes stronger due to an increase in the defect concentration connected to the need for charge compensation and not because of enhanced energy migration among the Mn^4+^ ions.

**Figure 4:**
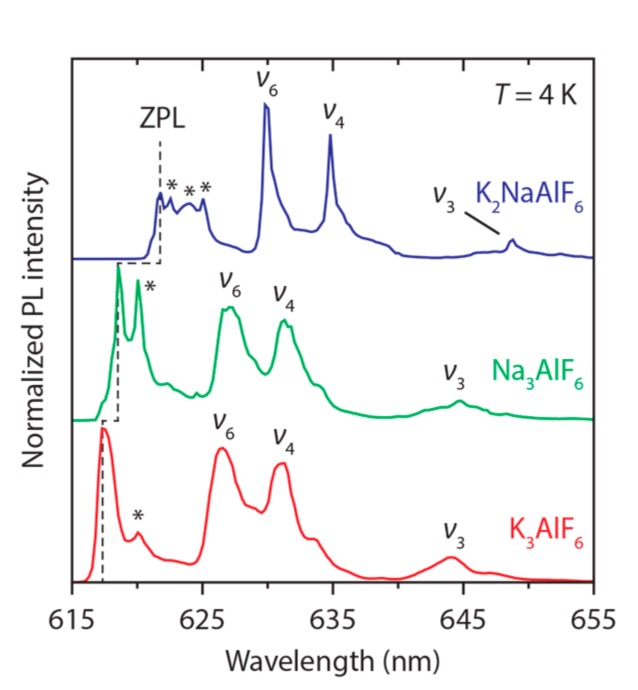
Emission spectra of K_3_AlF_6_:Mn^4+^ (1.2%) (red), Na_3_AlF_6_:Mn^4+^ (0.4%) (green) and K_2_NaAlF_6_:Mn^4+^ (0.9%) (blue) at *T* = 4 K. The excitation wavelength is 460 nm. Labels are assigned to the highest-energy zero-phonon line (ZPL) and vibronic ^2^E → ^4^A_2_ emissions (*ν*_3_, *ν*_4_ and *ν*_6_). The stars mark lines assigned to ZPLs of other Mn^4+^ sites (see text).

We performed low-temperature (*T* = 4 K) spectral measurements to get more insight into the Mn^4+^ sites in M_3_AlF_6_:Mn^4+^. In addition, the measurements at 4 K allow us to accurately compare the energy of the emitting ^2^E level in the different M_3_AlF_6_ hosts. Figure 4 displays emission spectra (*λ*_exc_ = 460 nm) at *T* = 4 K of M_3_AlF_6_:Mn^4+^. In line with the luminescence spectra at room temperature, the 4 K emission spectra of M_3_AlF_6_:Mn^4+^ consist of zero-phonon and vibronic ^2^E → ^4^A_2_ emission lines (labeled ZPL, *ν*_3_, *ν*_4_ and *ν*_6_). Multiple lines are observed in the ZPL region. The peaks marked with a star can be due to ^2^E → ^4^A_2_ electronic transitions that couple with low energy rotatory or translatory lattice vibrational modes [40,44]. Vibronic lines due to these modes are usually found at 50–150 cm^−1^ relative to the ZPL in emission spectra of Mn^4+^. Alternatively, the peaks marked with a star can be ZPLs of Mn^4+^ ions located on different sites than the Mn^4+^ ions yielding the most intense zero-phonon emission line (labeled ZPL in Figure 4). Mn^4+^ emission lines caused by lattice modes are typically very weak, and therefore it is more probable that the peaks marked with stars are ZPLs of Mn^4+^ ions with other geometric environments [33,44]. In addition, multiple Mn^4+^ sites can be expected, based on the need for charge compensation. Below, further evidence is given which indicates that the various sharp emission lines around 620 nm arise from MnF_6_ groups with different local geometries related to charge compensation.

**Figure 5:**
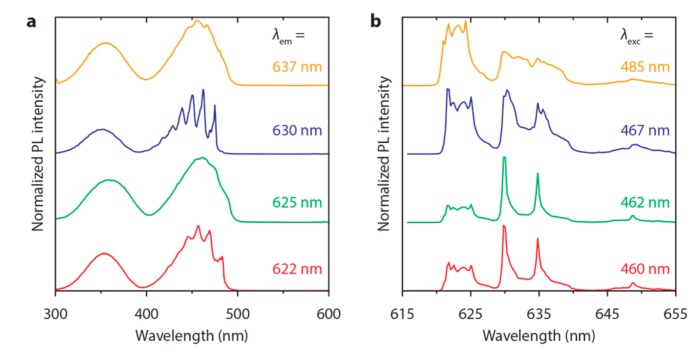
Emission and excitation spectra of K_2_NaAlF_6_:Mn^4+^ (0.9%) at *T* = 4 K. (**a**) Excitation spectra for *λ*_em_ = 622 nm (red), 625 nm (green), 630 nm (blue) and 637 nm (orange); (**b**) Emission spectra for *λ*_exc_ = 460 nm (red), 462 nm (green), 467 nm (blue) and 485 nm (orange).

In Figure 5 we investigate the presence of multiple Mn^4+^ sites by measuring 4 K luminescence spectra of K_2_NaAlF_6_:Mn^4+^ for various excitation and emission wavelengths. The excitation spectra in Figure 5a show that the structure in the ^4^A_2_ → ^4^T_2_ excitation band of K_2_NaAlF_6_:Mn^4+^ changes significantly with emission wavelength. If only one Mn^4+^ site would be present in K_2_NaAlF_6_:Mn^4+^, the excitation spectrum will have the same shape and structure for all emission wavelengths. However, here, the excitation spectrum changes significantly with emission wavelength, which indicates that more than one Mn^4+^ site is present in K_2_NaAlF_6_:Mn^4+^. Furthermore, the spectra in Figure 5b show that the shape of the ^2^E → ^4^A_2_ spectrum is different for various excitation wavelengths within the ^4^A_2_ → ^4^T_2_ band. This confirms that multiple Mn^4+^ sites exist in K_2_NaAlF_6_:Mn^4+^, and likely also in K_3_AlF_6_:Mn^4+^ and Na_3_AlF_6_:Mn^4+^. The presence of more than one Mn^4+^ site in M_3_AlF_6_:Mn^4+^ was not observed in previous reports on Mn^4+^-doped hexafluoraluminates [17,18]. Instead, from time-resolved measurements it was concluded that only one Mn^4+^ emission site was present in M_3_AlF_6_:Mn^4+^. The formation of geometrically different Mn^4+^ sites in M_3_AlF_6_:Mn^4+^ is expected as charge compensation is required for the ${\mathrm{Mn}}_{\mathrm{Al}}^{•}$ center. The charge compensating defect can be local or distant, i.e., in the first shell of cations around the Mn^4+^ ion or further away in the lattice. A distant defect will not influence the local geometry around the Mn^4+^ ion, whereas a local defect can cause a deformation of the fluorine octahedron around the Mn^4+^ ion. This will give rise to multiple differently charge compensated Mn^4+^ sites within the lattice, depending on the type and local geometry of charge compensation.

To study the influence of the M_3_AlF_6_ (M = Na, K) host on the energy of the Mn^4+2^E level, we compare the positions of the highest-energy ^2^E → ^4^A_2_ ZPLs in M_3_AlF_6_:Mn^4+^. The energies of these ZPLs (labeled ZPL in Figure 4) are 16,200 cm^−1^ (K_3_AlF_6_:Mn^4+^), 16,167 cm^−1^ (Na_3_AlF_6_:Mn^4+^) and 16,082 cm^−1^ (K_2_NaAlF_6_:Mn^4+^). The ^2^E level energies are also listed in Table 2. The energy of the ^2^E level for Mn^4+^ in M_3_AlF_6_ is in good agreement with the ^2^E level energy observed for Mn^4+^ in other fluoride hosts [45,46]. Furthermore, it is observed that the energy of the Mn^4+ 2^E level increases from K_2_NaAlF_6_ to Na_3_AlF_6_ to K_3_AlF_6_ (see dashed line in Figure 4). This indicates that the local structure and type of M^+^ cation in the second coordination sphere around the Mn^4+^ ion has an influence on the ^2^E level energy. Previous studies on M_2_SiF_6_:Mn^4+^ (M = Na, K, Rb or Cs) also show an influence of the M^+^ cation on the ^2^E level energy [33,40,44]. In these compounds the energy *E* of the ^2^E level follows the trend *E*(Na^+^) > *E*(K^+^) > *E*(Rb^+^) > *E*(Cs^+^), which suggests that the ^2^E level energy decreases with the radius or electron affinity of the M^+^ ion [21]. This is however not confirmed by our results for the M_3_AlF_6_:Mn^4+^ phosphors, where the highest ^2^E energy is found for K_3_AlF_6_:Mn^4+^. Instead, the results in Table 2 indicate that the energy of the ^2^E level increases when the Mn–F (Al–F) distance becomes longer or when the symmetry of the Mn^4+^ site (Al^3+^ site) is reduced. It is however not possible to draw definite conclusions from the data in Table 2 as the symmetry and distances in (part of) the MnF_6_ octahedra will change due to deformations caused by nearby charge compensating defects.

### 3.3. Thermal Quenching in M_3_AlF_6_:Mn^4+^

For high-power w-LED applications, the thermal quenching behavior of a phosphor is very important, as the temperature of the on-chip phosphor layer in a high-power w-LED reaches temperatures as high as 450 K. The thermal quenching behavior of K_3_AlF_6_:Mn^4+^ and Na_3_AlF_6_:Mn^4+^ has been investigated by Song et al. [17,18]. They reported that thermal quenching sets in around 423 K (150 °C) for K_3_AlF_6_:Mn^4+^ and Na_3_AlF_6_:Mn^4+^. More interestingly, they found that the integrated photoluminescence (PL) intensity of these phosphors doubles between room temperature and 423 K. This is a very useful property for high temperature applications, but is also very unexpected. For most Mn^4+^-doped fluorides, the PL intensity is relatively constant between room temperature and the temperature at which thermal quenching sets in [8,9,10].

**Figure 6:**
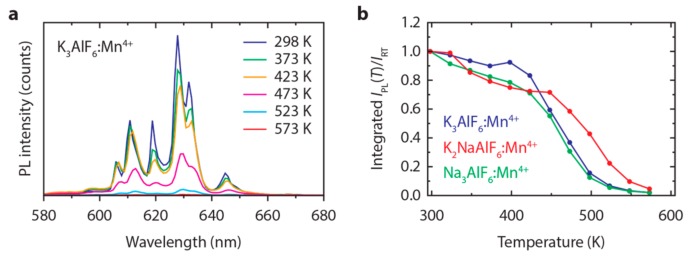
(**a**) Emission spectra of K_3_AlF_6_:Mn^4+^ (1.2%) at various temperatures between 298 K and 573 K (*λ*_exc_ = 450 nm); (**b**) Integrated PL intensity of K_3_AlF_6_:Mn^4+^ (1.2%) (blue), Na_3_AlF_6_:Mn^4+^ (0.4%) (green) and K_2_NaAlF_6_:Mn^4+^ (0.9%) (red) as a function of temperature between 300 and 600 K. The integrated PL intensity *I*_PL_ is scaled to the integrated PL intensity at room temperature *I*_RT_.

To investigate the thermal quenching of the Mn^4+^ emission in M_3_AlF_6_:Mn^4+^ (M = Na, K), we measure the integrated PL intensity of M_3_AlF_6_:Mn^4+^ as a function of temperature between 298 K and 600 K. Figure 6a shows emission spectra of K_3_AlF_6_:Mn^4+^ recorded in this temperature range. Emission spectra of Na_3_AlF_6_:Mn^4+^ and K_2_NaAlF_6_:Mn^4+^ between 298 K and 600 K can be found in Figure S1. The results show that the PL intensity of M_3_AlF_6_:Mn^4+^ slowly decreases up to 423 K (150 °C). Above this temperature, there is rapid quenching, with no emission intensity remaining at 573 K. After heating to 573 K, around half of the initial room-temperature PL intensity is retained. Part of the PL intensity is lost upon heating due to e.g., degradation of the phosphor, reduction/oxidation of the Mn^4+^ ions and formation of Mn-oxide impurities.

From the emission spectra recorded between 298 K and 600 K we obtain the temperature dependence of the integrated PL intensity (*I*_PL_), which is displayed in Figure 6b. The intensity is given relative to the integrated PL intensity at room temperature (*I*_RT_). The PL intensity gradually decreases between 300 K and 450 K, but then at higher temperatures rapidly drops due to an increased probability for non-radiative transitions from the ^2^E excited state (luminescence quenching). The luminescence quenching temperature *T*_½_, the temperature at which the PL intensity is half of its initial value, is around 460 K for K_3_AlF_6_:Mn^4+^ and Na_3_AlF_6_:Mn^4+^ and 485 K for K_2_NaAlF_6_:Mn^4+^. The *T*_½_ values fall in the range of quenching temperatures reported for Mn^4+^-doped fluoride phosphors [39,47]. The quenching temperatures of M_3_AlF_6_:Mn^4+^ are above the phosphor operating temperatures of high-power w-LEDs.

The temperature dependences we obtain for K_3_AlF_6_:Mn^4+^ and Na_3_AlF_6_:Mn^4+^ (Figure 6b) are significantly different from the work by Song et al. [17,18]. We observe a small decrease in the PL intensity between 298 K and 423 K, while they reported a doubling of the PL intensity between these temperatures. The decrease in PL intensity between 298 K and 423 K for M_3_AlF_6_:Mn^4+^ we ascribe to an increase of the energy transfer from Mn^4+^ ions to quenching sites (defects and impurities) with temperature [48]. The rapid quenching of the Mn^4+^ luminescence above 430 K is attributed to thermally activated crossing of the Mn^4+ 4^T_2_ excited state and ^4^A_2_ ground state, as is explained in [49].

**Figure 7:**
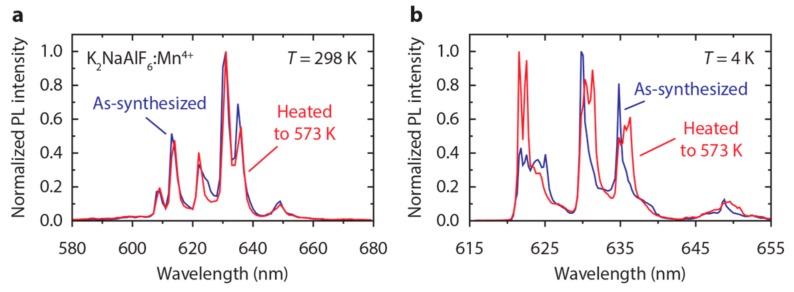
(**a**) Room-temperature emission spectra (*λ*_exc_ = 450 nm) of K_2_NaAlF_6_:Mn^4+^ (0.9%) for as-synthesized K_2_NaAlF_6_:Mn^4+^ phosphor (blue) and K_2_NaAlF_6_:Mn^4+^ phosphor that has been heated to 573 K (red); (**b**) Emission spectra of K_2_NaAlF_6_:Mn^4+^ (0.9%) at *T* = 4 K (*λ*_exc_ = 460 nm) for as-synthesized K_2_NaAlF_6_:Mn^4+^ phosphor (blue) and K_2_NaAlF_6_:Mn^4+^ phosphor that has been heated to 573 K (red).

Finally, we observe some interesting changes in the emission spectrum of K_2_NaAlF_6_:Mn^4+^ upon heating to 573 K. This is illustrated in Figure 7, which displays emission spectra recorded at *T* = 4 K and 298 K of K_2_NaAlF_6_:Mn^4+^ (0.9%) for as-synthesized K_2_NaAlF_6_:Mn^4+^ (blue spectra) and K_2_NaAlF_6_:Mn^4+^ phosphor that was heated to 573 K (red spectra). The room-temperature spectra in Figure 7a show that the structure of the ^2^E → ^4^A_2_ ZPL emission in K_2_NaAlF_6_:Mn^4+^ changes upon heating to 573 K. This effect is even more pronounced in the spectra measured at 4 K (Figure 7b). Four ZPLs of similar intensity are observed for the as-synthesized phosphor, while two ZPLs dominate the spectrum after heating at 573 K. In addition, the results in Figure 7b show that heating to 573 K changes the structure and intensity of the vibronic ^2^E → ^4^A_2_ emission lines. The changes in the emission spectra can be caused by a phase transition in the K_2_NaAlF_6_ crystal structure. However, the XRD patterns of as-synthesized K_2_NaAlF_6_:Mn^4+^ and K_2_NaAlF_6_:Mn^4+^ phosphor heated to 573 K both match the reference pattern of elpasolite K_2_NaAlF_6_, which indicates that no phase transition occurs (see Figure S2). We therefore explain the changes in the emission spectra upon high temperature annealing by a rearrangement of the Mn^4+^ sites in K_2_NaAlF_6_:Mn^4+^ upon heating to 573 K. Furthermore, the fact that two ZPLs dominate the emission spectrum of K_2_NaAlF_6_:Mn^4+^ after heating indicates that there is a redistribution in the abundance of different charge compensated Mn^4+^ sites. The results presented in Figure 7 show that post-synthesis heating can have a large effect on the luminescence properties of M_3_AlF_6_:Mn^4+^ and other Mn^4+^-doped fluoride phosphors.

## 4. Conclusions

Mn^4+^-doped fluorides have recently attracted considerable attention due to their potential for application in w-LEDs. For application in w-LEDs, it is important to understand and control the synthesis and luminescence properties of Mn^4+^-doped fluoride phosphors. Here, we report the synthesis of different M_3_AlF_6_:Mn^4+^ (M = Na, K) phosphors via a simple two-step co-precipitation method. Our synthesis method provides good control over Mn^4+^ doping and yields highly monodisperse ~20 μm smoothed octahedron shaped crystallites for K_2_NaAlF_6_:Mn^4+^, while irregularly shaped particles with a broad size distribution are obtained for K_3_AlF_6_:Mn^4+^ and Na_3_AlF_6_:Mn^4+^. All synthesized M_3_AlF_6_:Mn^4+^ phosphors show narrow red Mn^4+ 2^E → ^4^A_2_ luminescence that can be excited in the UV and blue spectral region. Luminescence spectra recorded at *T* = 4 K reveal that multiple Mn^4+^ sites are present in M_3_AlF_6_:Mn^4+^, which was not observed in previous reports. The presence of multiple Mn^4+^ sites is attributed to charge compensation that is required for Mn^4+^ on Al^3+^ sites. The results show that charge compensating defects have a large influence on the luminescence properties (e.g., spectra, QY, luminescence lifetime) of Mn^4+^-doped fluorides. Lowering of the QY by defect quenching is a problem for application of this class of Mn^4+^ phosphors. Finally, we investigated the thermal quenching behavior for M_3_AlF_6_:Mn^4+^. The luminescence quenching temperature of M_3_AlF_6_:Mn^4+^ is between 460 K and 490 K, which is above the phosphor operating temperature in high-power w-LEDs. If the QY can be improved by suppressing defect quenching, Mn^4+^-doped hexafluoroaluminates are a promising class of materials as their chemical and thermal stability is superior to the presently used commercial Mn^4+^ phosphors.

## Figures and Tables

**Table 1:** Amounts of Na_2_CO_3_, K_2_CO_3_, KF and 40% HF (aq) used in the synthesis of M_3_AlF_6_:Mn^4+^ (M = Na, K).

Phosphor	Na_2_CO_3_	K_2_CO_3_	KF	40% HF
Na_3_AlF_6_:Mn^4+^	15 mmol	-	-	15 mL
K_2_NaAlF_6_:Mn^4+^	5 mmol	10 mmol	-	15 mL
K_3_AlF_6_:Mn^4+^	-	-	40 mmol ^1^	3 mL

^1^ A 4:1 ratio of K:Al was used, as this gave K_3_AlF_6_ without impurity phases. With a 3:1 ratio, the obtained phosphor contained impurities of other crystal phases.

**Table 2:** Space group, Al^3+^ site symmetry and average Al–F distance for M_3_AlF_6_ (M = Na, K); zero-phonon line (ZPL) energy of the Mn^4+ 2^E → ^4^A_2_ emission in M_3_AlF_6_:Mn^4+^. Structural data obtained from Refs. [29,30,31].

Lattice	Space Group	Al^3+^ Symmetry	Al–F Distance (Å)	ZPL Energy (cm^−1^)
Na_3_AlF_6_	*P*2_1_/*n*	C_i_	1.808	16,167
K_2_NaAlF_6_	*Fm$\bar{3}$m*	O_h_	1.778	16,082
K_3_AlF_6_	*I*4_1_/*a*	C_1_	1.810	16,200

## Supplementary Materials

The following are available online at www.mdpi.com/1996-1944/10/11/1322/s1. Figure S1: Emission spectra of (**a**) Na_3_AlF_6_:Mn^4+^ (0.4%) and (**b**) K_2_NaAlF_6_:Mn^4+^ (0.9%) at various temperatures between 298 and 573 K (*λ*_exc_ = 450 nm); Figure S2: XRD patterns of K_2_NaAlF_6_:Mn^4+^ (2.9%) for as-synthesized K_2_NaAlF_6_:Mn^4+^ phosphor and K_2_NaAlF_6_:Mn^4+^ phosphor that has been heated to 573 K. The XRD patterns are in agreement with the reference diffraction pattern for K_2_NaAlF_6_ (PDF 01-072-2434, red); Video S1: Movie of synthesis of K_2_NaAlF_6_:Mn^4+^ phosphor. The phosphor shows bright red Mn^4+^ luminescence under 405 nm illumination.
