# Supplementary material for: Co-Precipitation Synthesis and Optical Properties of Mn4+-Doped Hexafluoroaluminate w-LED Phosphors
Source: Materials (Basel). 2017 Nov 17;10(11):1322. doi: 10.3390/ma10111322 (PMC5706269; doi:10.3390/ma10111322)
Supplement: Supplementary file 1 [file materials-10-01322-s001.zip › materials-234019-Supplementary Materials.pdf]

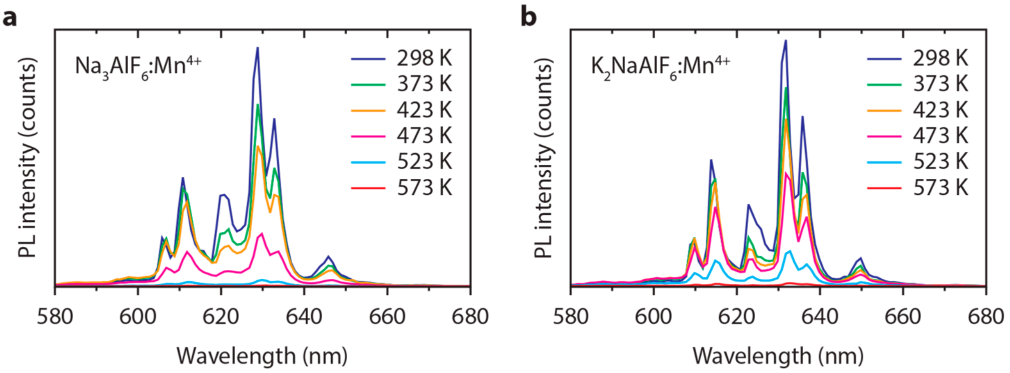

Figure S1. Emission spectra of (**a**) $Na_3AlF_6:Mn^{4+}$ (0.4%) and (**b**) $K_2NaAlF_6:Mn^{4+}$ (0.9%) at various temperatures between 298 and 573 K (λ_{exc} = 450 nm).

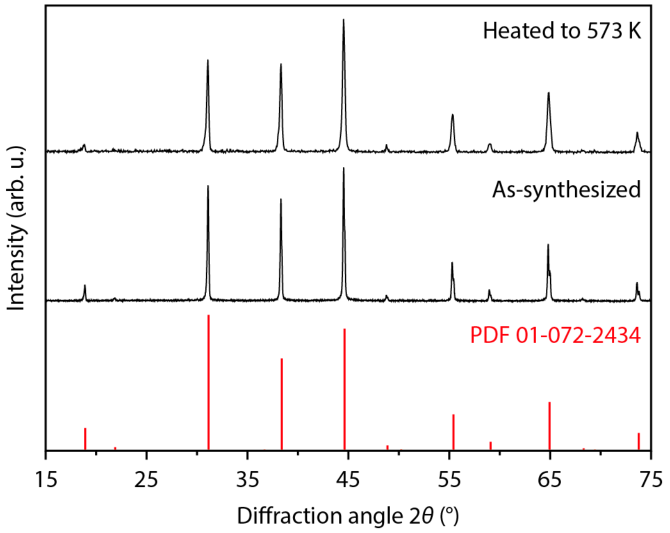

Figure S2. XRD patterns of $K_2NaAlF_6:Mn^{4+}$ (2.9%) for as-synthesized $K_2NaAlF_6:Mn^{4+}$ phosphor and $K_2NaAlF_6:Mn^{4+}$ phosphor that has been heated to 573 K. The XRD patterns are in agreement with the reference diffraction pattern for K_2NaAlF_6 (PDF 01-072-2434, red).

Video S1. Movie of synthesis of $K_2NaAlF_6:Mn^{4+}$ phosphor. The phosphor shows bright red Mn^{4+} luminescence under 405 nm illumination.
